# KAT6A/YAP/TEAD4 pathway modulates osteoclastogenesis by regulating the RANKL/OPG ratio on the compression side during orthodontic tooth movement

**DOI:** 10.1186/s40510-024-00530-6

**Published:** 2024-08-12

**Authors:** Kuang Tan, Jiayi Wang, Xinyu Su, Yunfei Zheng, Weiran Li

**Affiliations:** 1 Department of Orthodontics, Peking University School and Hospital of Stomatology, Beijing, 100081 China; 2 National Center for Stomatology, National Clinical Research Center for Oral Diseases, National Engineering Research Center of Oral Biomaterials and Digital Medical Devices, Beijing Key Laboratory of Digital Stomatology, NHC Key Laboratory of Digital Stomatology, NMPA Key Laboratory for Dental Materials, No.22, Zhongguancun South Avenue, Haidian District, Beijing, 100081 PR China

**Keywords:** KAT6A, Acetylated YAP, Osteoclastogenesis, The RANKL/OPG ratio, Orthodontic tooth movement

## Abstract

**Background:**

Orthodontic tooth movement (OTM) is a dynamic equilibrium of bone remodeling, involving the osteogenesis of new bone and the osteoclastogenesis of old bone, which is mediated by mechanical force. Periodontal ligament stem cells (PDLCSs) in the periodontal ligament (PDL) space can transmit mechanical signals and regulate osteoclastogenesis during OTM. KAT6A is a histone acetyltransferase that plays a part in the differentiation of stem cells. However, whether KAT6A is involved in the regulation of osteoclastogenesis by PDLSCs remains unclear.

**Results:**

In this study, we used the force-induced OTM model and observed that KAT6A was increased on the compression side of PDL during OTM, and also increased in PDLSCs under compression force in vitro. Repression of KAT6A by WM1119, a KAT6A inhibitor, markedly decreased the distance of OTM. Knockdown of KAT6A in PDLSCs decreased the RANKL/OPG ratio and osteoclastogenesis of THP-1. Mechanistically, KAT6A promoted osteoclastogenesis by binding and acetylating YAP, simultaneously regulating the YAP/TEAD axis and increasing the RANKL/OPG ratio in PDLSCs. TED-347, a YAP-TEAD4 interaction inhibitor, partly attenuated the elevation of the RANKL/OPG ratio induced by mechanical force.

**Conclusion:**

Our study showed that the PDLSCs modulated osteoclastogenesis and increased the RANKL/OPG ratio under mechanical force through the KAT6A/YAP/TEAD4 pathway. KAT6A might be a novel target to accelerate OTM.

**Supplementary Information:**

The online version contains supplementary material available at 10.1186/s40510-024-00530-6.

## Background

Orthodontic tooth movement (OTM) in alveolar bone is the dynamic equilibrium between osteogenesis of new bone and osteoclastogenesis of old bone, mediated by mechanical force [1]. A secure OTM depends on matched bone remodeling. The imbalance of bone formation and bone resorption, including the abnormality of mineral structure, bone volume, and bone growth, results in the difficulty of OTM [2, 3]. The periodontal ligament (PDL), a soft connective tissue between the root of the teeth and the alveolar bone, perceives and transmits mechanical signals to the alveolar bone during OTM. Multiple types of cells in PDL, including periodontal ligament stem cells (PDLSCs), monocyte/macrophage, fibroblast, etc., jointly maintain bone tissue homeostasis and mediate OTM under mechanical force [4].

PDLSCs are the primary stem cells in PDL, exhibiting multipotent differentiation properties, and can differentiate into osteoblasts. They respond to mechanical force by perceiving mechanical signals and producing a high level of cytokines, thus influencing other cells’ fate [5, 6]. The receptor activator nuclear factor κB ligand (RANKL) / osteoprotegerin (OPG) system is a classic regulator between osteoblasts and osteoclasts. RANKL is secreted by osteoblasts and stem cells, contributing to the formation of osteoclasts by binding to receptor activators of the nuclear factor κB (RANK) in pre-osteoclasts [7]. OPG acted as a decoy receptor by sequestering RANKL, interpreting RANKL’s interaction with RANK, termed an osteoclastogenesis inhibitory factor [8, 9]. An increase in the RANKL/OPG ratio often represents osteoclastogenesis in bone remodeling. Bone metabolism maintains homeostasis by continuously regulating the RANKL/OPG ratio [10, 11]. However, the inherent mechanism of PDLCSs regulating the RANKL/OPG ratio remains unknown.

YAP is a mechanical response protein that regulates cell differentiation under mechanical force [12]. VGLL4 is a YAP inhibitor, which promotes osteoblast differentiation and bone development by competitively binding with the transcriptional enhancer-associated domain 4 (TEAD4) [13]. In our previous study, we found that YAP played a part in the osteogenic differentiation of human bone mesenchymal stem cells (hBMSCs) by phase separation [14]. Knockdown of YAP or inhibition of the association of YAP with the TEAD family in bone marrow-derived macrophages (BMM) prevented the formation of osteoclasts [15]. Therefore, YAP is a crucial mediator of bone remodeling. However, the underlying mechanism of YAP regulating osteoclastogenesis in PDLSCs remains unknown.

KAT6A is a histone lysine acetyltransferase (also called MOZ and MYST3) belonging to the MYST family, reported to be enriched in the promoter of YAP, which contributed to the progression of hepatocellular carcinoma [16]. KAT6A can interact with and acetylate histones, playing roles in cell senescence and cancer development [17–19]. KAT6A can also acetylate nonhistone substrates, such as P53, COP1, and SMAD3, and function as an oncogene in malignant disease [20–22]. Recently, KAT6A was reported to regulate the stemness and osteogenic differentiation of hBMSCs [23]. A novel KAT6A mutation was dominant in bone marrow failure disease [24]. Hence, we suspect that KAT6A regulates cell differentiation by modulating YAP in PDLSCs.

In this study, a mechanical force-induced OTM model and a compressive force loading in vitro were applied to investigate the unique role of KAT6A in osteoclastogenesis. We found that KAT6A was universally upregulated in PDLSCs under mechanical stimuli. Repression of KAT6A decreased the distance of OTM and the osteoclastogenesis of THP-1. Mechanistically, KAT6A acetylated YAP and increased YAP binding to TEAD4, which promoted osteoclastogenesis by increasing the RANKL/OPG ratio. These results together indicated the importance of KAT6A in osteoclastogenesis during OTM and highlighted the potential of targeting KAT6A in accelerating OTM.

## Results

### KAT6A was significantly increased on the compression side of OTM

**Fig. 1:**
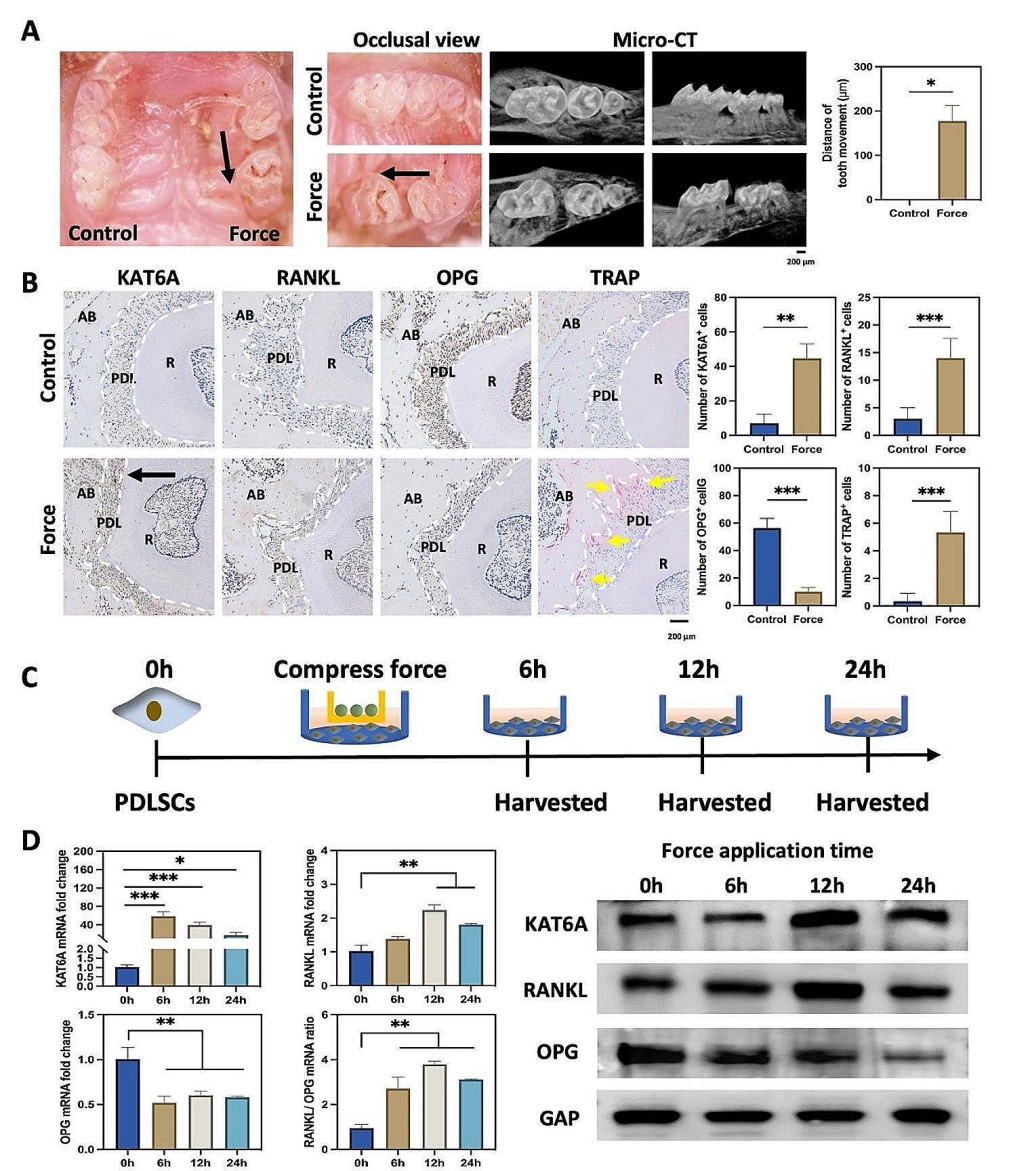
KAT6A was significantly increased on the compression side of OTM. (**A**) Representative occlusal view and micro-CT images of tooth movement after the mechanical force loading for 7 d. Quantitative analysis showed that the distance of tooth movement was increased after the mechanical force loading for 7 d (*n* = 5). The black arrow shows the direction of mechanical force. *P < 0.05 vs. control. Scale bar: 200 μm. (**B**) Representative immunohistochemistry images of KAT6A, RANKL, OPG, and TRAP on the control and the compression side of PDL. The number of KAT6A^+^ cells, RANKL^+^ cells, and TRAP^+^ cells was increased. The number of OPG^+^ cells was decreased in the periodontal tissues after force application (*n* = 3). AB means the alveolar bone; PDL means the periodontal ligament; R means the root. The black arrow shows the direction of mechanical force. The white dotted line indicates the shape of PDL. The yellow arrow points out the TRAP^+^ cells. **P < 0.01, ***P < 0.001 vs. control. Scale bar: 200 μm. (**C**) A schematic illustration shows the mechanical force loading in PDLSCs in vitro. (D) qPCR and western blot of KAT6A, RANKL, and OPG after mechanical force loading for 0 h, 6 h, 12 h, and 24 h. The mRNA and protein levels of KAT6A and the RANKL/OPG ratio were increased after mechanical force application (*n* = 3). *P < 0.05, **P < 0.01, ***P < 0.001 vs. 0 h

The experimental animal model of OTM was conducted. The distance of the first molar mesial movement was 177.4 ± 35.64 μm after mechanical force loading for 7 days (Fig. 1A and S1A). Immunochemistry staining showed that the shape of PDL was abnormal. The RANKL/OPG ratio was proven to regulate osteoclast differentiation [25]. On the compression side of OTM, RANKL-positive cells were increased while the OPG-positive cells were decreased, and the TRAP-positive osteoclasts were accumulated (Fig. 1B), indicating that osteoclast differentiation and bone resorption happened on the compression side. KAT6A is a histone acetyltransferase. We found that KAT6A-positive cells were increased universally after mechanical force application (Fig. 1B), implying that KAT6A was increased under mechanical force. Mechanical loading on PDLSCs in vitro showed that the mRNA of KAT6A was increased about 50-fold at 6 h and maintained an increase of approximately 30-fold and 20-fold at 12 h and 24 h. The protein level of KAT6A was also significantly increased at 12 h (Fig. 1D and S1C). Meanwhile, we detected the mRNA level of the other histone acetyltransferases as well, such as GCN5, P300, and PCAF. The results of qPCR showed that GCN5 was increased initially but decreased subsequently during mechanical stimuli, and P300 and PCAF were decreased after mechanical stimuli (Figure S1D). Thus, we investigated the role of KAT6A in this study. Furthermore, the RANKL/OPG ratio was increased at 6 h and peaked at 12 h, and the protein levels of the RANKL/OPG ratio peaked at 24 h (Fig. 1D and S1C). Overall, the data above indicated that KAT6A was significantly increased under compressive force in PDLSCs. KAT6A might regulate osteoclastogenesis by regulating the RANKL/OPG ratio.

### Inhibitor or knockdown of KAT6A inhibited osteoclastogenesis via regulating the RANKL/OPG ratio

**Fig. 2:**
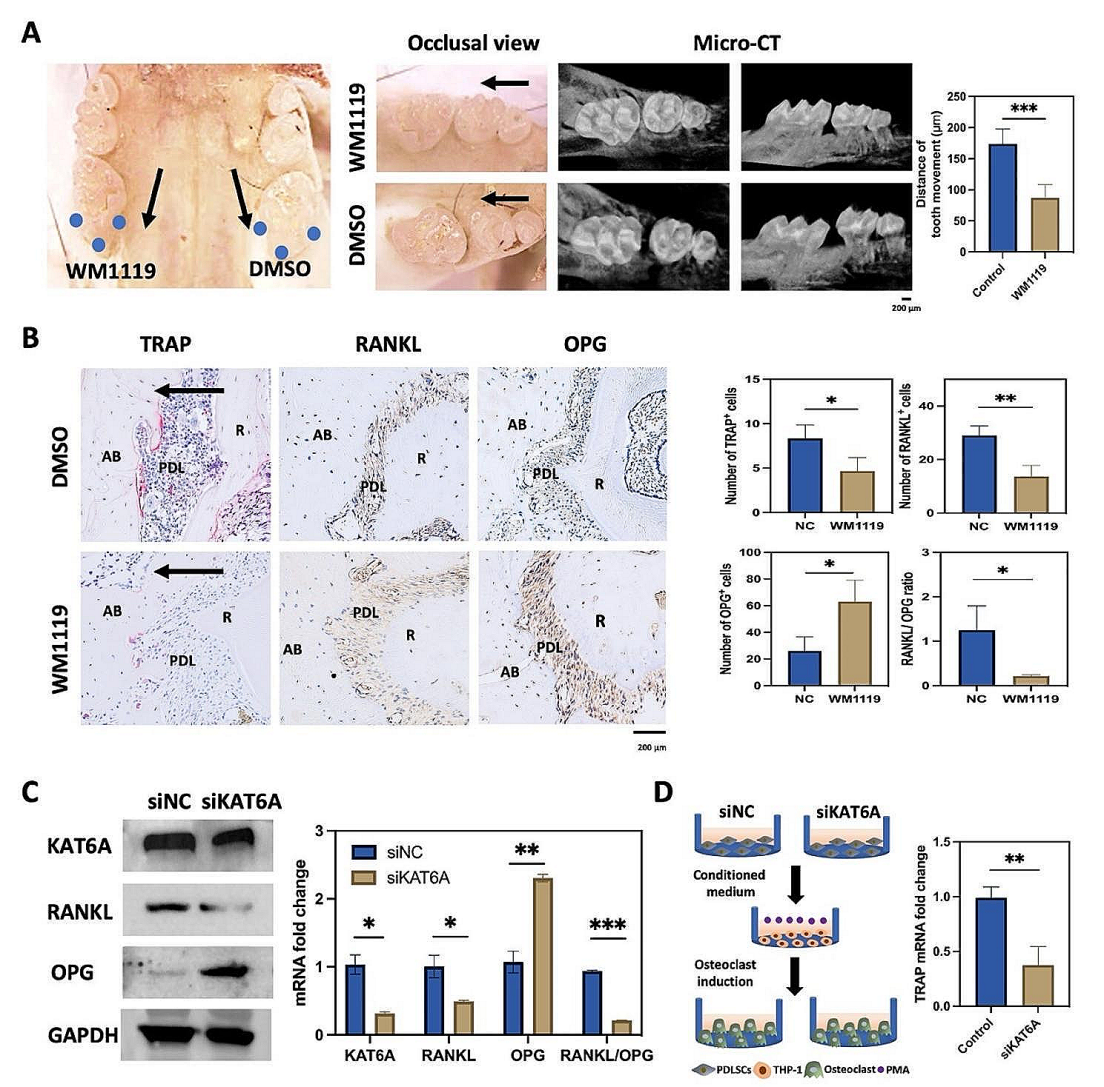
Inhibitor or knockdown of KAT6A inhibited osteoclastogenesis by regulating the RANKL/OPG ratio in PDLSCs. (**A**) Representative occlusal view and micro-CT images of tooth movement after the force loading for 7 d with periodontal injection of WM1119 or DMSO. Quantitative analysis showed that the distance of tooth movement was decreased after the force application for 7 d in the WM1119-treatment side (*n* = 5). The blue dots indicate the periodontal injection sites. The black arrow shows the direction of mechanical force. ***P < 0.001 vs. control. Scale bar: 200 μm. (**B**) Representative immunohistochemistry images of RANKL, OPG, and TRAP staining on the compression side of PDL with periodontal injection of WM-1119 or DMSO. The number of TRAP^+^ cells and RANKL^+^ cells were decreased. The number of OPG^+^ cells was increased in the periodontal tissues after WM1119 treatment (*n* = 3). AB means the alveolar bone; PDL means the periodontal ligament; R means the root. The black arrow shows the direction of mechanical force. *P < 0.05, **P < 0.01 vs. NC (DMSO). Scale bar: 200 μm. (**C**) qPCR and western blot of KAT6A, RANKL, and OPG after siKAT6A treatment. The mRNA and the protein levels of the RANKL/OPG ratio were decreased after siKAT6A treatment (*n* = 3). *P < 0.05, **P < 0.01, ***P < 0.001 vs. siNC. (**D**) The schematic of siKAT6A conditional medium culturing THP-1, and the qPCR of TRAP. The mRNA level of TRAP was decreased in the siKAT6A-treated group (*n* = 3). **P < 0.01 vs. control

To further investigate the role of KAT6A in osteoclastogenesis, we used WM1119, a KAT6A inhibitor, in the animal model and periodontal injected WM1119 (50 µg/kg) or DMSO at indicated sites (Fig. 2A). The distance of tooth mesial movement was significantly decreased on the WM1119-treatment side, and there were 173.8 ± 24.02 μm on the control side and 87.2 ± 20.95 μm on the WM1119-treatment side separately (Fig. 2A). Immunochemistry staining showed that the TRAP-positive cells and the RANKL-positive cells were decreased, and the OPG-positive cells were increased after injection of WM1119 (Fig. 2B), indicating that suppression of KAT6A influenced the RANKL/OPG ratio and prohibited osteoclast differentiation. To verify KAT6A modulating osteoclast differentiation in PDLSCs by regulating the RANKL/OPG ratio, knockdown experiments by siRNA were conducted. From the three targets of siKAT6A, we selected siKAT6A-2097 in the following study for its highest knockdown efficiency (Figure S2). qPCR and western blot showed that the RANKL/OPG ratio was decreased in PDLSCs after siKAT6A treatment (Fig. 2C and S2B). Furthermore, conditional medium culturing PDLSCs with or without knockdown of KAT6A was added to culture THP-1. The mRNA level of TRAP indicated that the knockdown of KAT6A significantly reduced the osteoclast differentiation of THP-1 (Fig. 2D). These results collectively suggested that KAT6A inhibited bone resorption by decreasing the RANKL/OPG ratio under mechanical force.

### KAT6A regulated the expression and the acetylation of YAP

**Fig. 3:**
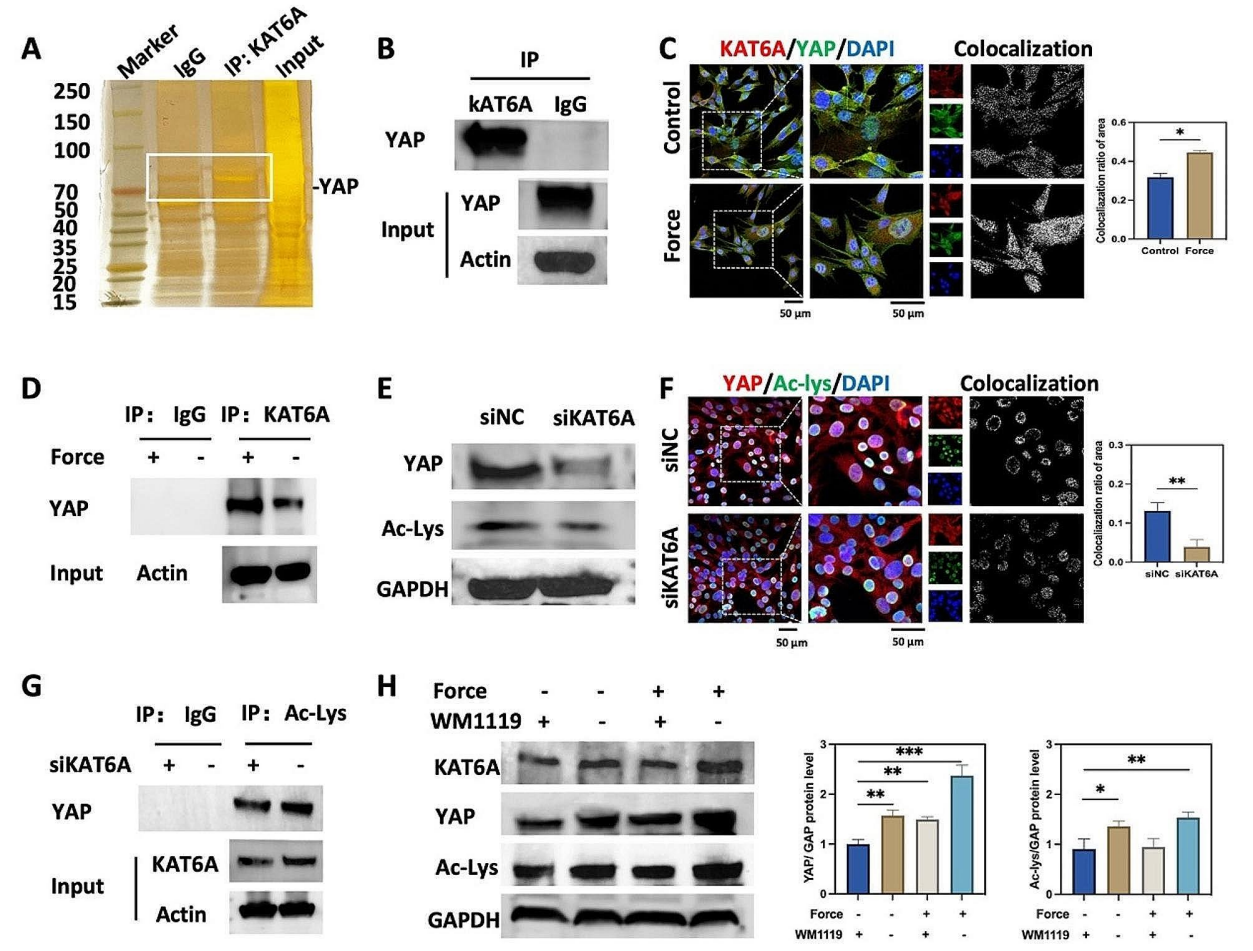
KAT6A regulated the expression and the acetylation of YAP. (**A**) Silver staining of SDS-PAGE gel of KAT6A-associated proteins in PDLSCs. The area of the white box was analyzed by mass spectrometry. (**B**) CO-IP was performed to detect KAT6A binding with YAP (*n* = 3). (**C**) Representative immunofluorescence images of KAT6A (red) and YAP (green) in PDLSCs. The fluorescence colocalization area of YAP and KAT6A was increased after mechanical force loading (*n* = 3). Scale bar: 50 μm. (**D**) CO-IP was performed to detect KAT6A binding with YAP after mechanical force loading. The protein band indicated that the mechanical force increased KAT6A binding with YAP (*n* = 3). (**E**) The western blot of YAP and pan-acetylation after siKAT6A treatment. (**F**) Representative immunofluorescence images of YAP (red) and pan-acetylation (green) in PDLSCs. The fluorescence colocalization area of YAP and pan-acetylation was decreased after siKAT6A treatment (*n* = 3). **P < 0.01 vs. siNC. Scale bar: 50 μm. (**G**) CO-IP was performed to detect the acetylated YAP after siKAT6A treatment. The protein band indicated that the knockdown of KAT6A decreased the acetylated YAP (*n* = 3). (**H**) The western blot of KAT6A, YAP, and pan-acetylation. And the quantitative analysis of YAP and pan-acetylation in PDLSCs. The protein level of YAP and acetylation level was upregulated after mechanical force loading, which could partly be reversed by WM-1119 (24 h, 25 µM, *n* = 3). *P < 0.05, **P < 0.01, ***P < 0.001 vs. control

We next investigated the mechanism of KAT6A modulating bone resorption during OTM. As demonstrated by previous studies, KAT6A regulated YAP in hepatocellular carcinoma cell lines HepG2 and Huh7 [16]. YAP was also essential for bone remodeling [15]. Thus, we hypothesized that KAT6A modulated bone remodeling by regulating YAP. We performed mass spectrometry (MS) to verify our hypothesis and identified KAT6A-binding partners. Figure 3A and Table S1 showed that YAP was a partner with KAT6A. To support this result, we performed co-immunoprecipitation (CO-IP) and found that endogenous KAT6A could bind with YAP in PDLSCs (Fig. 3B). Double immunostaining showed that KAT6A and YAP had overlapped localization. Moreover, after mechanical stimuli, the colocalization ratio of KAT6A and YAP was increased (Fig. 3C). The intensity of YAP binding to KAT6A also increased after mechanical stimuli (Fig. 3D). These results indicated that the mechanical force reinforced KAT6A in combination with YAP.

KAT6A, an acetyltransferase known to acetylate with both histones and nonhistones [22, 26], was the focus of our study. We aimed to understand its effect on YAP acetylation. Our findings, as depicted in Fig. 3E and S2C, indicated that the knockdown of KAT6A led to a decrease in YAP expression and the overall acetylation level, which suggested that KAT6A played a role in modulating the expression of YAP and the pan-acetylation. To further investigate the mechanism of KAT6A regulating YAP, we conducted double immunostaining of YAP and pan-acetylation, as well as CO-IP, by using an anti-pan-acetylation antibody after the knockdown of KAT6A. As shown in Fig. 3F, YAP and pan-acetylation partly overlapped, and the western blot confirmed that YAP could be immunoprecipitated by an anti-pan-acetylation antibody (Fig. 3G). Furthermore, the colocalization ratio of YAP and pan-acetylation, as well as the intensity of YAP immunoprecipitated by an anti-pan-acetylation antibody, were found to be decreased after siKAT6A treatment, which suggested that the knockdown of KAT6A decreased the acetylation level of YAP. Furthermore, WM1119 was found to partly attenuate the force-induced elevation of YAP and pan-acetylation levels (Fig. 3H). These results underscored the physical association of KAT6A with YAP and its role in regulating the expression and the acetylation of YAP.

### Mechanical force increased the expression of YAP and TEAD4

**Fig. 4:**
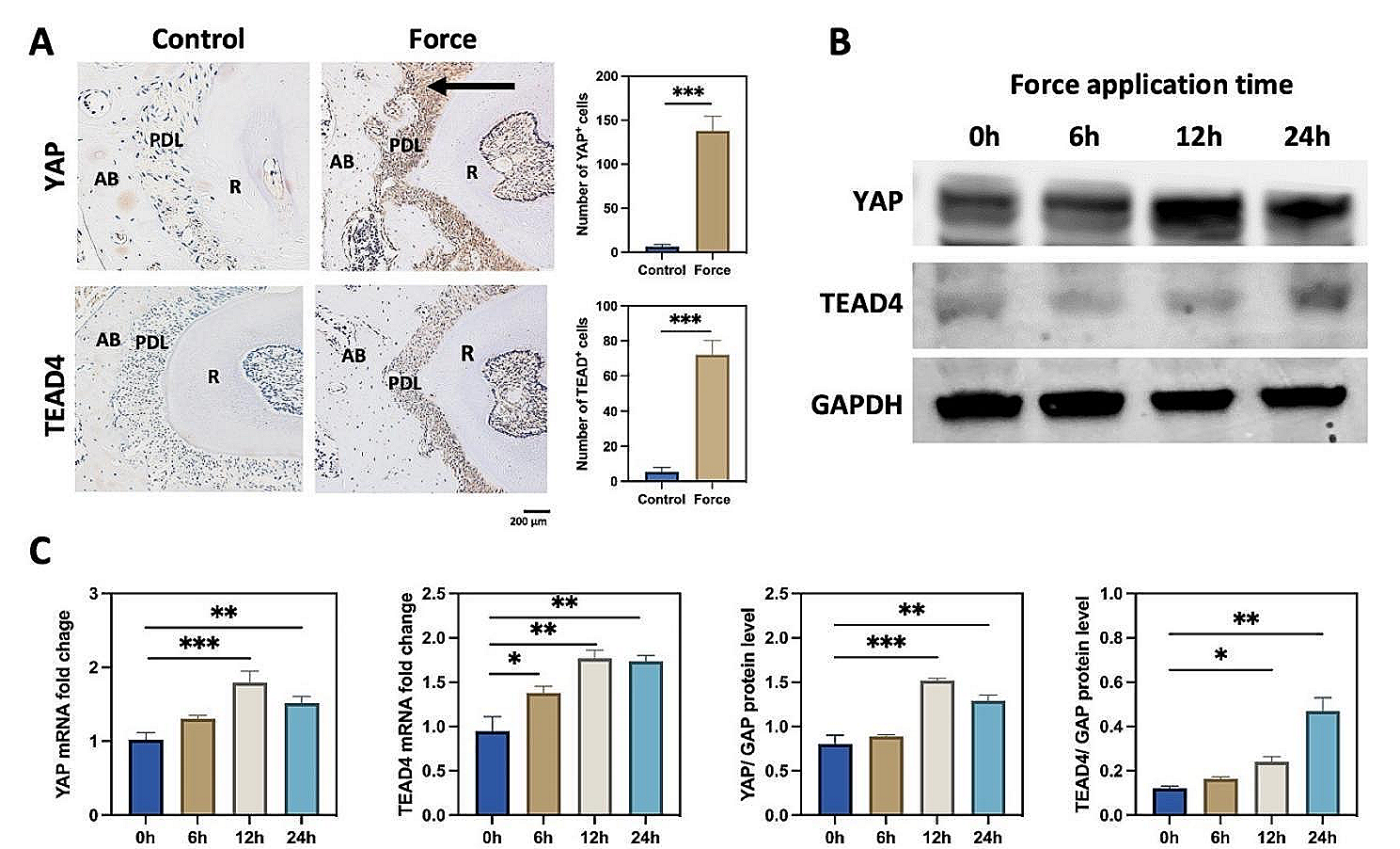
Mechanical force increased the expression of YAP and TEAD4. (**A**) Representative immunohistochemistry images of YAP and TEAD4 in compression side of PDL. The number of YAP^+^ and TEAD4^+^ cells was increased on the compression side of PDL (*n* = 3). The black arrow shows the direction of mechanical force. AB means the alveolar bone; PDL means the periodontal ligament; R means the root. ***P < 0.001 vs. control. Scale bar: 200 μm. (**B**) The western blot of YAP and TEAD4 after mechanical force application for 0 h, 6 h, 12 h, and 24 h. (**C**) The protein and mRNA levels of YAP and TEAD4 after mechanical force application in vitro (*n* = 3). *P < 0.05, **P < 0.01, ***P < 0.001 vs. 0 h

As mentioned in Fig. 3H, we found that YAP was upregulated in PDLSCs under mechanical force. TEAD4 functions as a YAP-targeted protein in the Hippo pathway [27]. Thus, we further investigated the role of YAP and TEAD4 in mechanical force-induced bone resorption. Immunochemistry staining revealed that YAP and TEAD4 were increased on the compression side during OTM (Fig. 4A). Western blot and qPCR showed that the expression of YAP was significantly enhanced at 12 h, and TEAD4 was increased at 12 h and 24 h during mechanical force application in vitro (Fig. 4B and C). These data above suggested that YAP and TEAD4 were upregulated after mechanical stimulation and might mediate bone resorption during the OTM.

### Mechanical force increased YAP association with TEAD4

**Fig. 5:**
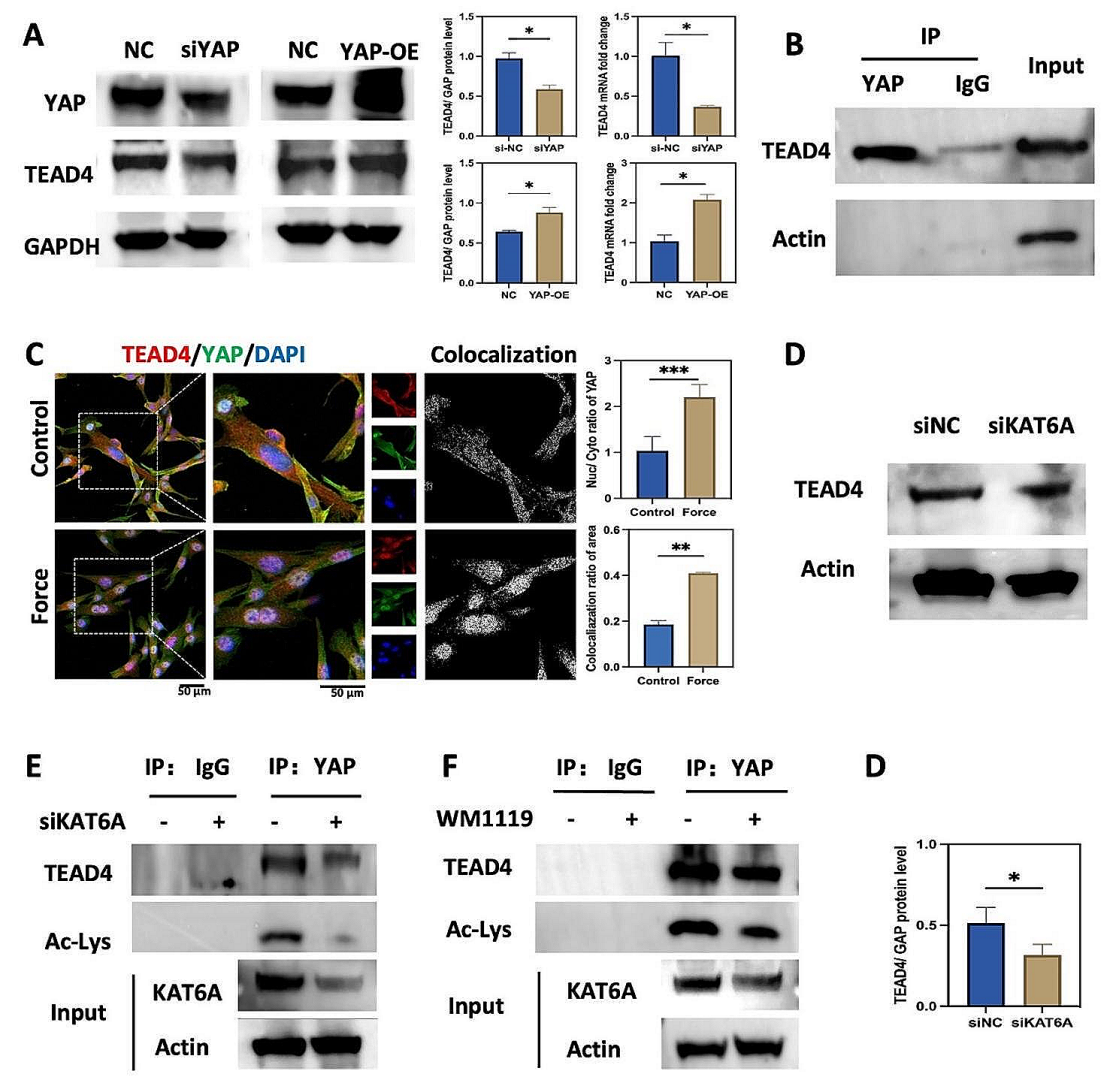
Mechanical force increased YAP association with TEAD4. (**A**) qPCR and western blot of TEAD4 after siYAP or YAP lentivirus treatment in PDLSCs. The protein and mRNA levels of TEAD4 were decreased and increased after siYAP or YAP lentivirus treatment, respectively (*n* = 3). *P < 0.05 vs. control. (**B**) CO-IP was performed to detect YAP binding with TEAD4. (**C**) Representative immunofluorescence images of TEAD4 (red) and YAP (green) in PDLSCs. The nuc/cyto of YAP and the fluorescence colocalization area of YAP and TEAD4 were increased after mechanical force loading (*n* = 3). Scale bar: 50 μm. **P < 0.01, ***P < 0.001 vs. control. (**D**) Western blot of TEAD4 in PDLSCs. The protein level of TEAD4 was decreased after siKAT6A treatment (*n* = 3). *P < 0.05 vs. siNC. (**E**) and (**F**) CO-IP was performed to detect YAP binding with TEAD4 and pan-acetylation after siKAT6A or WM-1119 treatment (24 h, 25 µM). The protein band indicated that the knockdown or inhibition of KAT6A decreased YAP association with TEAD4 and the pan-acetylation (*n* = 3)

TEAD4 was reported to play a role in cell differentiation, dependent on or independent of YAP [13, 15]. However, whether the effect on osteoclastogenesis induced by TEAD4 is dependent on YAP in PDLSCs remains unclear. Thus, we investigated the relationship between YAP and TEAD4 in PDLSCs under mechanical force. siYAP-1676 and YAP lentivirus were transfected into PDLSCs to knock down and overexpress YAP, respectively (Figs. S3A and S3B). Western blot and qPCR showed that the knockdown and overexpression of YAP downregulated and upregulated TEAD4 in PDLSCs accordingly (Fig. 5A), implying that YAP regulated the expression of TEAD4. Moreover, CO-IP showed that the TEAD4 could be immunoprecipitated by endogenous YAP (Fig. 5B), and the double staining of YAP and TEAD4 also indicated that YAP in combination with TEAD4 in PDLSCs (Fig. 5C). YAP was reported to function as a transcription co-activator and would translocate into nuclear, promoting the transcription of the downstream gene [28]. Immunofluorescence staining indicated that the YAP’s nuclear/cytoplasm (nuc/cyto) ratio was increased under mechanical force (Fig. 5C), indicating that YAP was activated under mechanical stimuli. Moreover, the colocalization ratio between YAP and TEAD4 was increased after mechanical stimuli (Fig. 5C), indicating that the combination of YAP and TEAD4 was reinforced by mechanical force. These results implied that YAP and TEAD4 were activated after mechanical stimuli, and the mechanical force increased the binding between YAP and TEAD4.

Interestingly, we found the knockdown of KAT6A decreased the expression of TEAD4 as well (Fig. 5D). Thus, we further probe the mechanism of KAT6A modulating TEAD4. siKAT6A and WM1119 were used respectively to investigate how KAT6A regulated TEAD4. The knockdown or repression of KAT6A significantly interfered with the interaction between YAP and TEAD4 and downregulated the pan-acetylation binding with YAP (Fig. 5E and F). Thus, we inferred that KAT6A regulated the combination of the YAP and TEAD4, the acetylated YAP, which might influence the expression of TEAD4.

### The YAP/TEAD4 axis modulated osteoclastogenesis by regulating the RANKL/OPG ratio under mechanical force

**Fig. 6:**
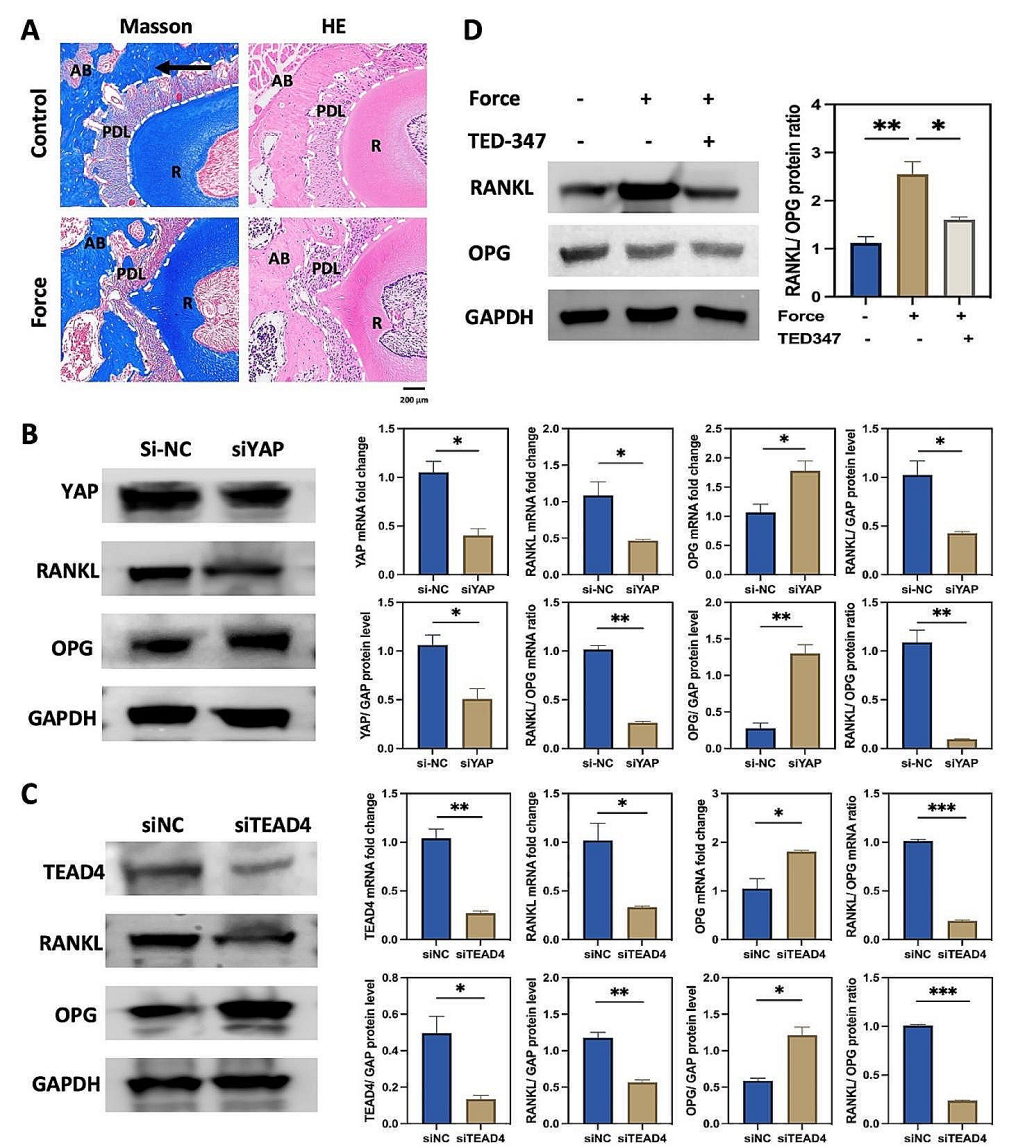
The YAP/TEAD4 axis modulated osteoclastogenesis by regulating the RANKL/OPG ratio under mechanical force. (**A**) Representative Masson and H&E staining of the compression side of PDL. The black arrow shows the direction of mechanical force. The white dotted line indicates the shape of PDL. AB means the alveolar bone; PDL means the periodontal ligament; R means the root. Scale bar: 200 μm. (**B**) and (**C**) qPCR and western blot of RANKL and OPG after siYAP and siTEAD4 treatment in PDLCSs. The protein and mRNA levels of the RANKL/OPG ratio were decreased after siYAP or siTEAD4 treatment (*n* = 3). *P < 0.05, **P < 0.01 ***P < 0.01vs. siNC. **D**. Western blot and the quantitative analysis of the RANKL/OPG ratio in PDLSCs. The protein level of the RANKL/OPG ratio was upregulated after mechanical force loading, which could partly be reversed by TED-347 (24 h, 5 µM, *n* = 3). *P < 0.05, **P < 0.01 vs. the force group

Masson and HE staining showed that the PDL was compressed on the compression side during OTM (Fig. 6A). Our previous data demonstrated that KAT6A was upregulated and modulated osteoclast differentiation by regulating the RANKL/OPG ratio under mechanical stimuli (Figs. 1B and D and 2B and D). KAT6A also regulated the expression of YAP and TEAD4 (Figs. 3E and 5D). Thus, we want to verify whether YAP and TEAD4 regulate osteoclastogenesis by modulating the RANKL/OPG ratio. We knocked down and overexpressed YAP and TEAD4 in PDLSCs, respectively. Consistent with our assumption, the knockdown of YAP or TEAD4 decreased the RANKL/OPG ratio (Fig. 6B and C). Overexpression of YAP or TEAD4 increased the RANKL/OPG ratio (Figures S3C and S4C). Moreover, compressive force in vitro upregulated the RANKL/OPG ratio in PDLSCs, which was reversed by simultaneous treatment with TED-347, a YAP-TEAD4 interaction inhibitor (Fig. 6D). These data showed that the YAP/TEAD4 axis was vital to bone resorption via regulating the RANKL/OPG ratio. Inhibiting the interaction between YAP and TEAD4 could decrease the RANKL/OPG ratio.

**Fig. 7:**
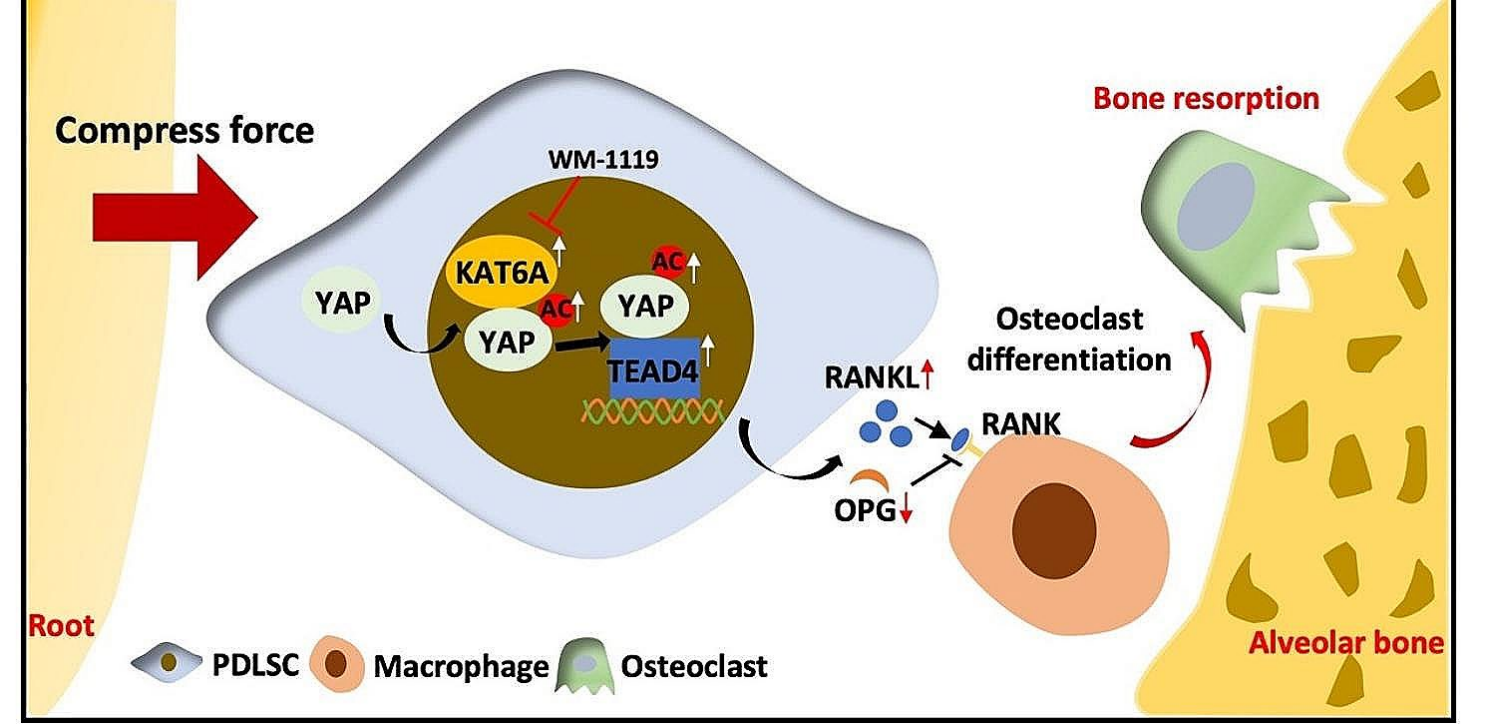
Schematic illustration of the KAT6A/YAP/TEAD4 pathway modulating osteoclastogenesis during OTM by regulating the RANKL/OPG ratio

Taken together, our results revealed that the KAT6A/YAP/TEAD4 pathway was activated in PDLSCs under mechanical force, thus affecting the RANKL/OPG ratio and promoting osteoclastogenesis during the OTM (Fig. 7).

## Discussion

OTM is the equilibrium of bone resorption mediating by osteoclastogenesis and bone formation mediating by osteogenesis, depending on the suitable mechanical force and the regulation of multiple cells [29]. As the primary mesenchymal stem cells (MSCs) in the PDL, PDLSCs respond to mechanical force to maintain periodontal tissue homeostasis and alveolar bone remodeling [30]. However, the underlying mechanism of PDLSCs regulating bone resorption remains unclear. In this study, we discovered a new KAT6A/YAP/TEAD4 pathway in PDLSCs, which might be a potential pathway affecting bone resorption and OTM. Mechanistically, KAT6A was significantly upregulated after mechanical stimuli. Then, KAT6A bound and acetylated YAP, which promoted YAP association with TEAD4. Activating the KAT6A/YAP/TEAD4 pathway could increase the RANKL/OPG ratio, thus promoting osteoclastogenesis. Pharmacologic repression of KAT6A by WM1119 or knockdown of KAT6A by siRNA markedly inhibited osteoclast differentiation of THP-1 and OTM when applied for mechanical force (Fig. 7).

KAT6A is a lysine acetyltransferase and can acetylate histone and nonhistone [22, 26]. Previous studies have demonstrated that KAT6A played an essential role in developing malignant diseases, such as leukemia, lung cancer, breast cancer, etc [31–33]. Recently, a few studies have reported KAT6A’s roles in stemness and osteogenic differentiation of hBMSCs [23]. A KAT6A mutation has been associated with bone marrow failure disease [24]. However, the role and mechanism of KAT6A in osteoclastogenesis during OTM are still unknown. Here, our data demonstrated that KAT6A was critical for osteoclastogenesis. High expression of KAT6A accelerated bone resorption by increasing the RANKL/OPG ratio in PDLSCs. Targeting KAT6A could be a promising strategy to accelerate OTM.

YAP is a critical effector in the Hippo pathway [34]. Phosphorylation is the most common post-translation modification of YAP [35]. When the Hippo pathway is activated, YAP is phosphorylated at Ser127, inhibiting YAP’s nuclear localization and transcriptional activity. When the Hippo pathway is closed, YAP is dephosphorylated and translocated into nuclear as a transcription coactivator, thus activating the downstream transcription factor [36, 37]. The acetylation cycle of YAP is also devoted to the activation and nuclear accumulation of YAP. Feng et al. reported that the increase of acetylated YAP was accompanied by nuclear accumulation of YAP, which inhibited the progress of FLT3-ITD + leukemia [38]. Some studies also reported that the acetylated YAP functioned in atherosclerosis and responded to DNA damage [39, 40]. However, the acetylation modification of YAP in bone resorption remains elusive. Our data identified YAP as a new nonhistone substrate of KAT6A, which advanced our knowledge of the function of KAT6A and YAP and their association with bone resorption. One deficiency that should be pointed out in our study was that we need to identify the acetylation modification site of YAP. Further, we will clarify the detailed mechanism of acetylation modification of YAP in regulating bone remodeling. Moreover, the interaction between KAT6A and its nonhistone acetylated substrates, such as p53 and SMAD3, was enhanced by the phosphorylation of the substrate [18, 22]. Thus, we will investigate the relationship between phosphorylation modification and acetylation modification of YAP when YAP functions as the substrate of KAT6A.

TEAD4, a downstream target protein of YAP in the Hippo pathway, has been implicated in tumor progression and osteoclast differentiation of BMM [41, 42]. However, whether YAP regulates TEAD4 in PDLSCs and influences osteoclastogenesis during OTM remains unclear. Our research revealed that the mechanical force increased YAP’s interaction with TEAD4 (Fig. 5C) and suggested that the acetylated YAP might be involved in the increased expression of TEAD4 (Fig. 5D and F). Modifying YAP’s acetylation might be a new method for YAP to regulate TEAD4.

OTM includes bone resorption on the compression side and bone formation on the tension side. Our study explored the role of KAT6A on the compression side during the OTM. However, PDLSCs on the tension side may have different phenotypes and biological responses [43]. The reaction of KAT6A on the compression or tension sides may also be different. The comparison of KAT6A on the compression and tension sides will be investigated in the future. We will also perform on KAT6A knockout mice to further confirm the function of KAT6A in bone remodeling and OTM.

## Conclusions

In summary, our study reveals that the KAT6A is increased in PDLSCs under mechanical force, contributing to osteoclastogenesis during OTM. We also provide new insight into the posttranslational modification of YAP, enlarging the function of KAT6A as acetyltransferase of nonhistone protein, and acetylated YAP may be a transcription co-activator regulating TEAD4 in PDLSCs. These results suggest a novel KAT6A/YAP/TEAD4 pathway during OTM, indicating the importance of KAT6A in osteoclastogenesis by regulating the RANKL/OPG ratio. This study also implies that KAT6A dysfunction may interfere with the the mechanical force-induced OTM.

## Methods

### Animals

Male 6-7-week-old, wild-type mice (C57BL/6), were used in this study. C57BL/6 mice were obtained from Weitong Lihua company (Beijing, China). The animals were kept under pathogen-free conditions. All animal experimental procedures were approved by the Peking University Animal Care and Use Committee (LA2020329) and conformed to the ARRIVE guidelines.

### Force-induced OTM model and the intervention group

The force-induced OTM model was built as a previous study [44]. Nickel-titanium coil springs (0.2 mm thickness, Smart technology) were ligated from the incisors to the maxillary first molar (Figures S1A and S1B), with the contralateral maxillary first molar serving as the control. Each coil spring can provide a constant force of ~ 30 g to move teeth. The incisors were bonded using flowable resin (3 M ESPE, St. Paul, MN, USA). To investigate the role of KAT6A in bone resorption. WM1119 (50 µg/kg, HY-102058, MedChemExpress, USA) was used at 0, 2, 4, and 6 days on the intervention side, dimethyl sulfoxide (DMSO, D2650, Sigma-Aldrich, USA) of the same volume were used in the control side. Periodontal injection of WM1119 or DMSO at indicated sites in the compression side of the first molar during the OTM (three blue dots indicated the periodontal injection sites in Fig. 2A). After 7 days, the springs were removed, and the mice were sacrificed; the maxillae were harvested for further study. A stereomicroscope (SWZ1000, Nikon, Japan) was used to observe tooth movement from the occlusal view.

### Micro-computed tomography (Micro-CT)

Mice maxillae were fixed in 4% paraformaldehyde (PFA, Aoqing Biotechnology, China) for 24 h at 4 °C. The tooth movement was scanned by micro-CT(Bruker Skyscan, Belgium) at a resolution of 10 μm. The CT data were imported into NRecon and CTvox software to get the 3D reconstructed images. A computer-assisted design software, Data Viewer and CTAn (Bruker), were applied to get the distance of tooth movement. The distance between the first molar and the second molar was measured from the midpoint of the distal-marginal ridge of the first molar to the midpoint of the mesial-marginal ridge of the second molar. The average of two measurements was calculated as the tooth movement distance.

### Hematoxylin and eosin (H&E), Masson, tartrate-resistant acid phosphatase (TRAP) staining and analyses

After fixation in 4% PFA at 4 °C for 24 h, the samples were decalcified in ethylenediaminetetraacetic acid (15%, pH 7.4) for 14 d. Then, the specimens were dehydrated. Consecutive 4 μm-thick sections were obtained from the compression side and the control side. H&E, Masson, and TRAP staining were performed as previously described [14, 44]. Briefly, the sections were treated with EDTA antigen retrieval (C1034, Solarbio, Beijing) for 10 min at 37 °C. Then, 5% goat serum (ZLI-9021, ZSGB-BIO, China) was used to block nonspecific binding. Then, the sections were stained with an H&E staining kit (GP1031, Servicebio, China), Masson staining kit (GP1032, Servicebio, China), and TRAP staining kit (387 A-1KT; Sigma, USA). TRAP-positive multinucleated (> 3 nuclei) cells attached to the alveolar bone surface were counted (*n* = 5).

### Immunohistochemical staining and analyses

Immunohistochemical staining was performed as previously described [14]. Briefly, sections were blocked in 5% goat serum (ZLI-9021, ZSGB-BIO, China) and then incubated with primary antibodies. The primary antibodies including anti-KAT6A (1: 200, AB 2761888, ABclonal, China), anti-YAP (1: 200, 13584-1-AP, Proteintech, China), anti-TEAD4 (1: 200, ab155244, Abcam, USA), anti-RANKL (1: 200, 66610-1-Ig, Proteintech, China) and anti-OPG (1: 200, PA3284, Abmart, China) were added to sections at 4℃ overnight. After rinsing three times with phosphate-buffered saline (PBS), the sections were incubated with corresponding horseradish peroxidase-conjugated secondary antibodies (ZB-2306, ZSGB-Bio, China) at room temperature for 1 h. DAB kit (ZLI-9018, ZSGB Bio, China) was used for coloration. The samples were observed under an upright microscopy (Olympus BX60, Japan). The quantitative analyses using Image J software (National Institutes of Health, USA).

### Cell culture

PDLSCs were obtained from premolar teeth from healthy patients for orthodontic extraction and were cultured according to previously published protocols [45, 46]. All research protocols were approved by the Ethics Committee of the Peking University School of Stomatology (PKUSSIRB-201837096). Informed consent was obtained from all patients involved. Briefly, the extracted teeth were repeatedly washed with sterile PBS containing 10% penicillin/streptomycin (15140-122, Gibco, USA). Then, PDL tissues were scraped from the root and digested in collagenase (17100-017, Gibco, USA) and trypsin (25300-054, Gibco, USA) for 1 h at 37˚C. Cultured medium consisting of α-modified Eagle’s medium (α-MEM, Gibco, USA), 10% fetal bovine serum (CB14808348, Gibco, USA), and 1% penicillin/streptomycin (Gibco) was used to culture cells at 37˚C with 5% CO2. One week later, primary cells migrated outward from PDL tissues and were passaged using trypsin (Gibco) at 80- 90% confluence.

The following procedures were used for cell cryopreservation and thawing. Briefly, cells were digested by trypsin (Gibco) and then centrifuged. Then cell pellets were mixed with serum-free cryopreservation (C40100, NCM Biotech, China) and preserved at -80 °C. Thawing cells were water bathed at 37 °C, then the cell suspensions were added to the cultured medium. PDLSCs were expanded at a split ratio of 1: 2 − 1: 3, and the passages 3–7 were used for this study.

To investigate the role of KAT6A in PDLSCs. WM1119 (25 µM), a KAT6A inhibitor, was added to the medium. To inhibit the interaction between YAP and TEAD4, TED-347 (5 µM, HY-125,269, MedChemExpress, USA) was added to the medium. For the control group, DMSO (Sigma-Aldrich) of the same volume was added to the medium.

THP-1 monocytes were purchased from Procell company (CL-0233, China). Cultured medium consisting of RPMI-1640 (Gibco, USA), 10% fetal bovine (Gibco), and 1% penicillin/streptomycin (Gibco). Before inducing osteoclast differentiation, THP-1 was treated with phorbol 12-myristate 13-acetate (PMA; 100 ng/ml, Sigma-Aldrich, USA) for 24 h. Then RANKL (50 ng/mL, 310-01, Peprotech) and macrophage colony-stimulating factor (M-CSF, 30 ng/mL, 300 − 25, Peprotech) were added to the medium every 2 days for osteoclast induction.

### Application of mechanical stress

PDLSCs were seeded into 6-well plates, and mechanical force loading in vitro was applied as previously described [47]. Briefly, when the cell density reached 80-90%, a cover glass and metallic balls (2 g/ cm^2^) were placed on the top of the cell layer for 0, 6, 12, and 24 h respectively. PDLSCs in the control group were cultured without compressive force. The “force” group in vitro represented mechanical force loading for 12 h.

### Conditional culture of THP-1

To investigate the role of KAT6A in osteoclastogenesis. Conditional medium culturing PDLSCs with or without knockdown KAT6A were mixed with the cultured medium culturing THP-1 (v/v = 1: 1). RANKL (Peprotech) and M-CSF (Peprotech) were simultaneously added to the medium to induce osteoclast differentiation of PMA-treated THP-1. After inducing for 5 days, the cells were harvested. The method detecting mRNA of TRAP was described in section of “Total RNA extraction and quantitative real-time PCR (qPCR)”.

### Total RNA extraction and quantitative real-time PCR (qPCR)

Total RNA was extracted from PDLSCs using TRIzol reagent (15596026, Invitrogen, USA) according to the manufacturer’s instructions. A cDNA Reverse Transcription Kit (AG11728, Accurate Biology, China) was used to synthesize cDNA from 1 µg of total RNA. The SYBR Green Master Mix (AG1176, Accurate Biology, China) was used for qRT-PCR on ABI 7500 (Applied Biosystem). Thermal settings were as follows: 95 °C for 30 s, 40 cycles of 95 °C for 5 s, and 60 °C for 30 s. The mean of the housekeeping gene glyceraldehyde3-phosphate dehydrogenase (GAPDH) acted as an internal reference. q-PCR was performed three times. The primers used in the experiment are listed in Table S2, and the results were analyzed using the method of − ΔΔCt as previously described [48].

### Western blot

Total protein from PDLSCs was extracted using RIPA buffer (P0013K, Beyotime, China) mixed with protease and phosphatase inhibitor cocktail (78426, Thermo, USA). 4-20% gradient sodium dodecyl sulfate-polyacrylamide (SDS-PAGE) gel (ET12420, ACE Biotechnology, China) was used to separate equal quantities of proteins. Then, the proteins were transferred onto polyvinylidene difluoride (PVDF) membranes (Merck Millipore, USA). After a blocking incubation with 5% milk solution, the PVDF membranes were incubated overnight at 4 °C with primary antibodies as follow: anti-KAT6A (1: 1000, ABclonal), anti-YAP (1: 1 000, Proteintech), anti-TEAD4 (1: 1000, Abcam), anti-RANKL (1: 1000, Proteintech), anti-OPG (1: 1000, Abmart), anti-pan-acetylation (1: 1000, 66289-1-Ig, proteintech, China), anti-GAPDH (1: 1000, 60004-1-Ig, Proteintech, China) and anti-β-actin (1: 1000, 66009-1-Ig, Proteintech, China). Then, the PVDF membranes were washed with TBST three times. The corresponding secondary antibodies (ZSGB-BIO, China) were used to incubate the membranes for 1 h. After washing three times with TBST, an imaging system (Bio-Rad) and the ImageJ software (NIH) were used to evaluate and quantify the intensities of the protein bands.

### Co-IP assay

CO-IP assay was performed as previously described [14]. Briefly, 100 µL of IP lysis buffer (containing 1× protease inhibitor) was added to cells for 30 min followed by centrifugation at 10,000 g for 20 min. 30 µL of protein A-Sepharose bead slurry was prepared for each sample. For cell lysis, samples in the experimental group were mixed with 5 µg of anti-KAT6A (ABclonal), 5 µg of anti-YAP (Proteintech), or 5 µg of anti-pan-acetylation (Proteintech); those in the negative control group were mixed with 5 µg of control IgG (30000-0-AP, Proteintech, China). Incubation buffer (200 µL) and protein A Sepharose beads (30 µL) were incubated in spin columns overnight at 4 °C. The supernatant was discarded, and the pellet was washed five times with 800 µL of 1× washing buffer. The pellet complex was eluted in 80 µL of elution buffer and mixed with 10 µL of alkali neutralization buffer and 23 µL of 5× loading buffer. Samples were boiled for 10 min and subjected to western blotting as described in section of “Western blot”.

### Mass spectrometric analyses

SDS-PAGE gels were stained with the Silver Staining kits (P0017S, Beyotime, China). Proteomics analyses for KAT6A-associated proteins were performed by Peking University Health Science Center (Beijing, China). Briefly, the KAT6A-associated protein of PDLSCs was collected as described in section of “Co-IP assay”. Then, the protein samples were analyzed by LC-MS/MS using the Triple Quad 7500 system (SCIEX, USA). The raw data were processed by the software of Proteome Discoverer. The raw data were searched against the UniProt database. The KAT6A-interacting proteins of control and KAT6A groups selected Score Sequest HT ≥ 1.5, deleting contaminating proteins and removing negative reference proteins of the IgG group.

### Small interfering RNA (siRNA) transfection

siRNA targeting KAT6A (si-KAT6A-1320, si-KAT6A-2097 and si-KAT6A-2388), targeting YAP (si-YAP-1876, si-YAP-1536 and siYAP-1676), targeting TEAD4 (siTEAD4-339, siTEAD4-742 and siTEAD4-1431) and the scramble control (si-NC) were purchased from GenePharma company (Shanghai, China). The siRNA sequences are listed in Table S3. When the degree of cell fusion reached 70-80%, cells were transfected with siRNA by opti-MEM (Gibco, USA) and Lipofectamine 2000 (Lipo2000, Invitrogen, USA) according to the manufacturer’s instructions. Briefly, 10 µl siRNA and 3 µl Lipo2000 were mixed with 125 µl Opti-MEM separately for 5 min. Then the compound of siRNA-Opti-MEM and lipo2000-Opti-MEM were mixed gently and incubated for 20 min at room temperature. The compound was added to the culturing medium without antibiotic and then the medium was changed in 24 h. After 48 h, total RNA or protein was extracted as described in sections of “Total RNA extraction and quantitative real-time PCR (qPCR)”.

### Lentivirus transfection

YAP and TEAD4 lentivirus were purchased from Mijia Biotech (Beijing, China). The lentivirus transfection was conducted according to the manufacturer’s instructions. Briefly, when the degree of cell fusion reached 70–80%, cells were transfected with lentivirus for 24 h. Then, change the medium with puromycin (10 µg/ml) every 2 days for three times. Total RNA or protein was extracted as described in sections of “Total RNA extraction and quantitative real-time PCR (qPCR)” and “Western blot”.

### Immunofluorescence staining

PDLSCs were fixed in 4% PFA (Aoqing Biotechnology) for 15 min at room temperature and permeabilized in 0.2% (v/v) Triton X-100 (9002-93-1, BioRuler, USA) for 10 min. Cells were washed with PBS. A block step was done in 10% goat serum (ZSGB-BIO) for 30 min. Cells incubated overnight at 4 °C with primary antibodies as follow: anti-KAT6A (1: 200, ABclonal), anti-YAP (1: 200, Proteintech), anti-TEAD4 (1: 200, Abcam) and anti-pan-acetylation (1: 200, Proteintech). Then cells were washed with PBS three times. Cells were incubated with secondary antibodies for 1 h at room temperature. The secondary antibodies used were as follows: Alexa Fluor-488 labeled goat anti-mouse IgG (H + L) (1: 200, SA00013-1, Proteintech, China) and Alexa Fluor-594 labeled goat anti-rabbit IgG (H + L) (1: 200, SA00013-4, Proteintech, China). Nuclei were stained with DAPI (C0065, Solarbio, Beijing). Images were captured under a confocal microscope (Leica, TCS-SP8 STED 3X, Germany). Fluorescence intensity or co-localization of the proteins was analyzed and quantified by Image J software (USA).

### Statistical analysis

All experiments were conducted at least in triplicate, and the results are presented as the mean ± standard deviation (SD). The two-tailed unpaired Student’s t-test was used for two-group comparisons. One-way analysis of variance (ANOVA) was applied for comparison of three or more groups. All statistical analyses were carried out by GraphPad Prism version 9 (GraphPad Software Inc., USA). The threshold of statistical significance was set as *p* < 0.05.

### Electronic supplementary material

Below is the link to the electronic supplementary material.

Supplementary Material 1

## Data Availability

The data that support the findings of this study are available from the corresponding author upon reasonable request.

## Abbreviations

OTM: Orthodontic tooth movement
PDLCSs: Periodontal ligament stem cells
PDL: Periodontal ligament
RANKL: Receptor activator nuclear factor κB ligand
OPG: Osteoprotegerin
RANK: Receptor activators of the nuclear factor κB
TEAD: Transcriptional enhancer-associated domain
hBMSCs: Human bone mesenchymal stem cells
MS: Mass spectrometry
MSCs: Mesenchymal stem cells
Micro-CT: Micro-computed tomography
H&E: Hematoxylin and eosin
TRAP: Tartrate-resistant acid phosphatase
qPCR: Quantitative real-time PCR
Co-IP: Co-immunoprecipitation
siRNA: Small interfering RNA
