# Supplementary material for: KAT6A/YAP/TEAD4 pathway modulates osteoclastogenesis by regulating the RANKL/OPG ratio on the compression side during orthodontic tooth movement
Source: Prog Orthod. 2024 Aug 12;25:29. doi: 10.1186/s40510-024-00530-6 (PMC11317454; doi:10.1186/s40510-024-00530-6)
Supplement: Supplementary file 1 — Supplementary Material 1 [file 40510_2024_530_MOESM1_ESM.docx]

**Supplementary Material**

**KAT6A/YAP/TEAD4 pathway modulates** **osteoclastogenesis by regulating the RANKL/OPG ratio on the compression side during orthodontic tooth movement**


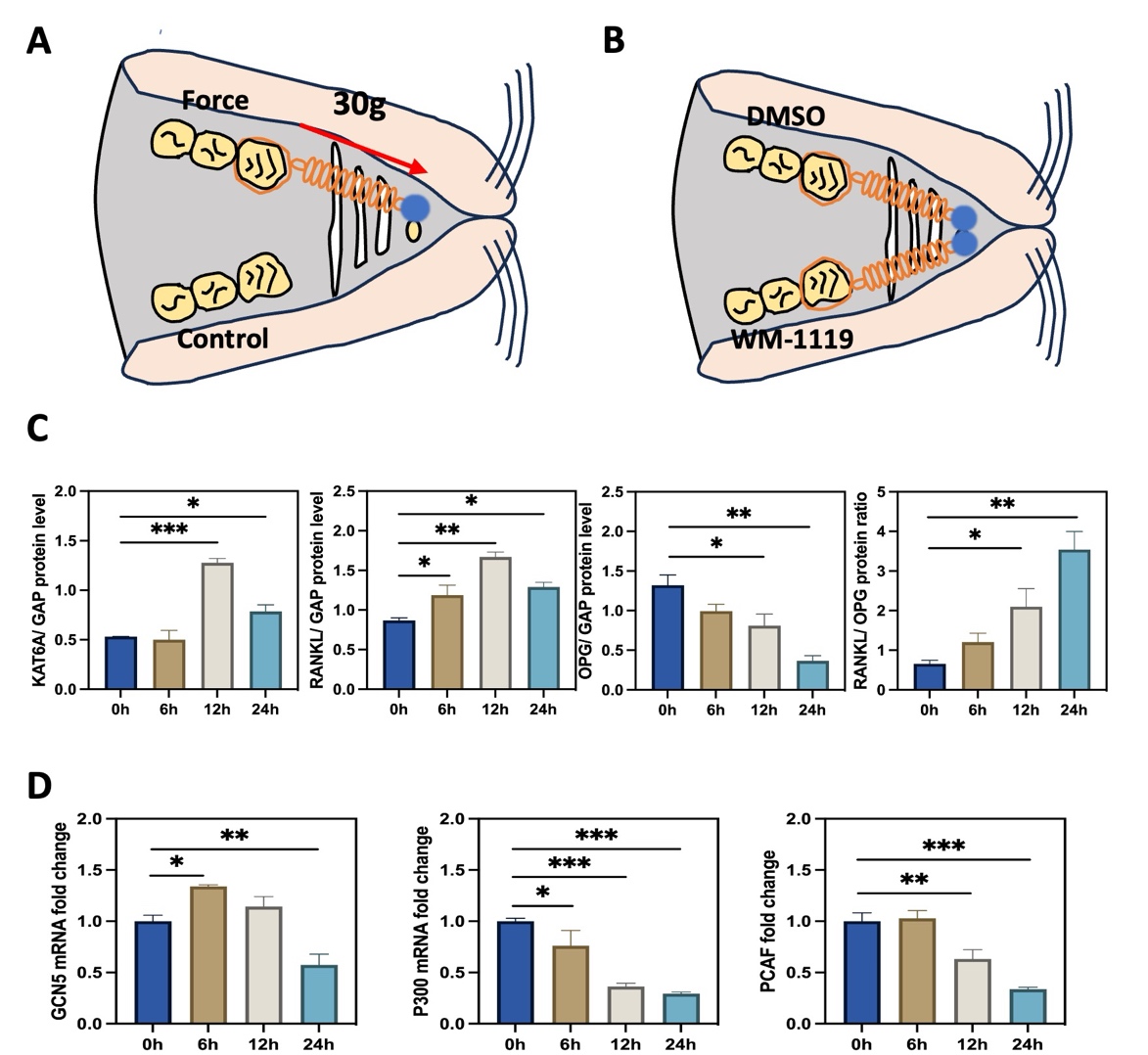


**Figure S1** A. A schematic illustration of unilateral mechanical loading during OTM. B. A schematic illustration of bilateral mechanical loading with periodontal injection of WM-1119 or DMSO during OTM. C. The quantitative analysis of the protein. The protein level of KAT6A and the RANKL/OPG ratio were increased after mechanical stimuli (n = 3). D. The mRNA level of GCN5, P300, and PCAF in PDLSCs after mechanical stimuli (n = 3). *P < 0.05, **P < 0.01, ***P < 0.001 vs. 0 h.


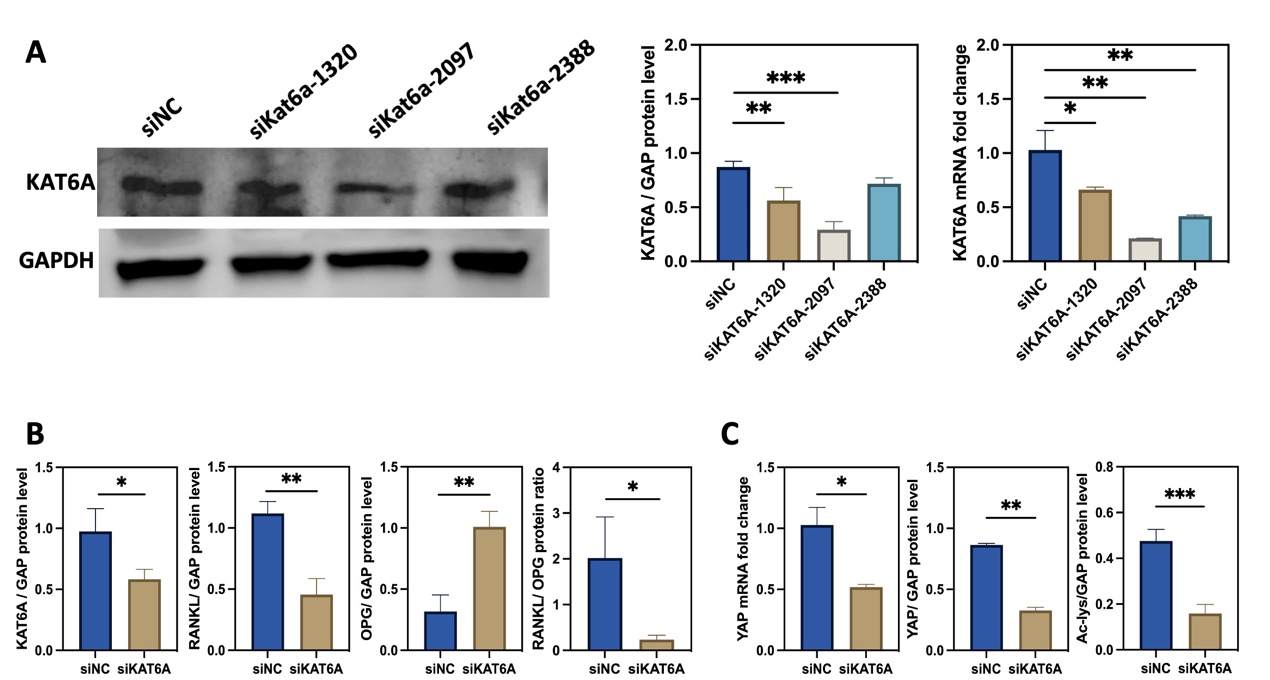


**Figure S2** A. The knockdown efficiency of siKAT6A. qPCR and western blot showed that the siKAT6A-2097 possessed the most highly knockdown efficiency (n = 3). *P < 0.05, **P < 0.01, ***P < 0.001 vs. siNC. B. The quantitative analysis of the protein. The protein level of the RANKL/OPG ratio was decreased after siKAT6A treatment (n = 3). *P < 0.05, **P < 0.01 vs. siNC. C. The quantitative analysis of the protein and the mRNA. The protein and mRNA levels of YAP were decreased after siKAT6A treatment in PDLSCs (n = 3). *P < 0.05, **P < 0.01 vs. siNC.


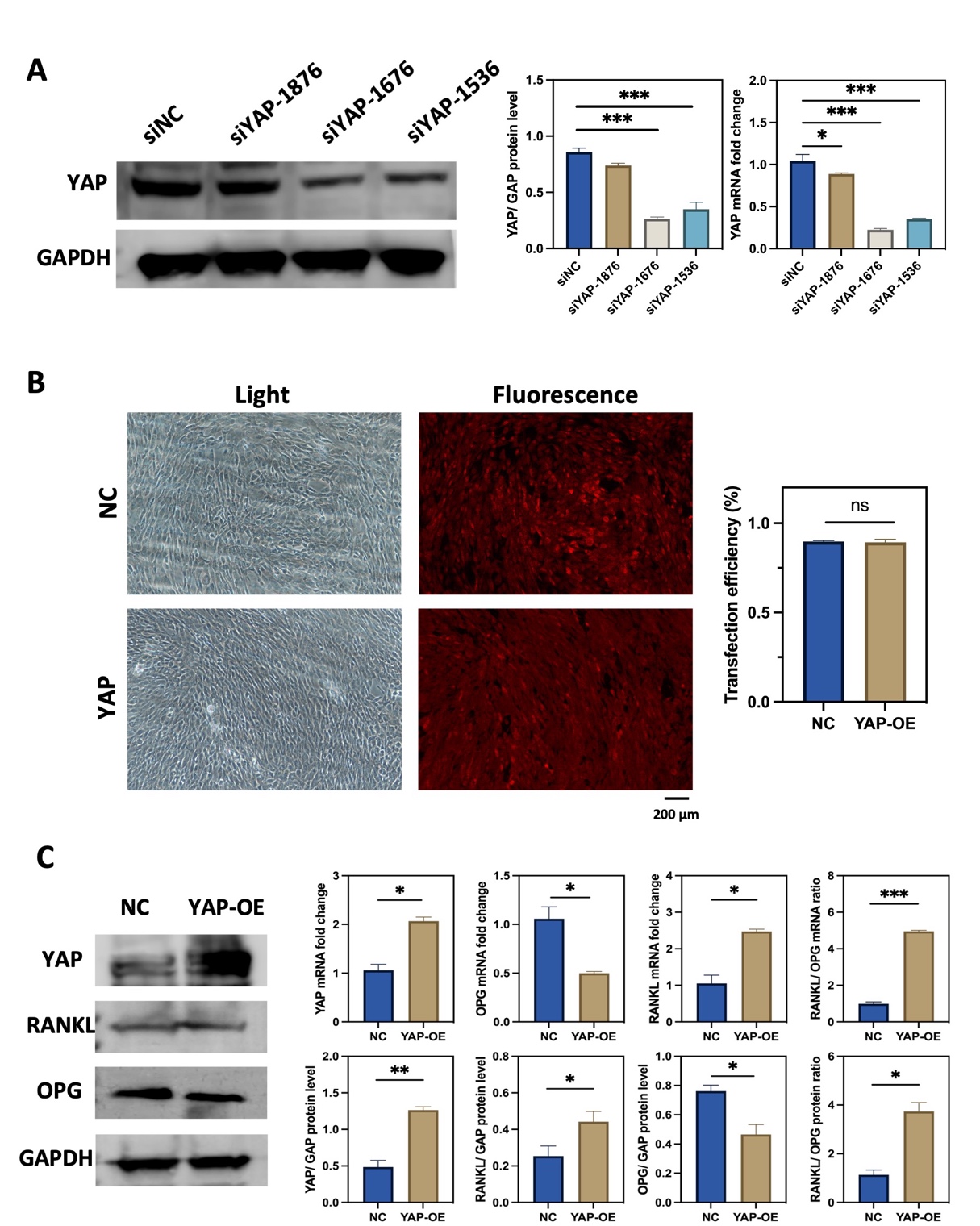


**Figure S3** The knockdown and overexpression of YAP in PDLSCs. A. The knockdown efficiency of siYAP. qPCR and western blot showed that the siYAP-1676 possessed the most highly knockdown efficiency (n = 3). *P < 0.05, ***P < 0.001 vs. siNC. B. Representative fluorescence images of the overexpression of YAP lentivirus. NC was transfected by RFP lentivirus. Quantitative analysis showed that the transfection efficiency of YAP and RFP lentivirus had no significant difference (n = 3). ns: no significant difference vs. NC. Scale bar: 200 μm. C. qPCR and western blot of RANKL and OPG after overexpression of YAP lentivirus in PDLCSs. The protein and mRNA levels of the RANKL/OPG ratio were increased after overexpression of YAP (n = 3). *P < 0.05, **P < 0.01, ***p < 0.001 vs. NC.


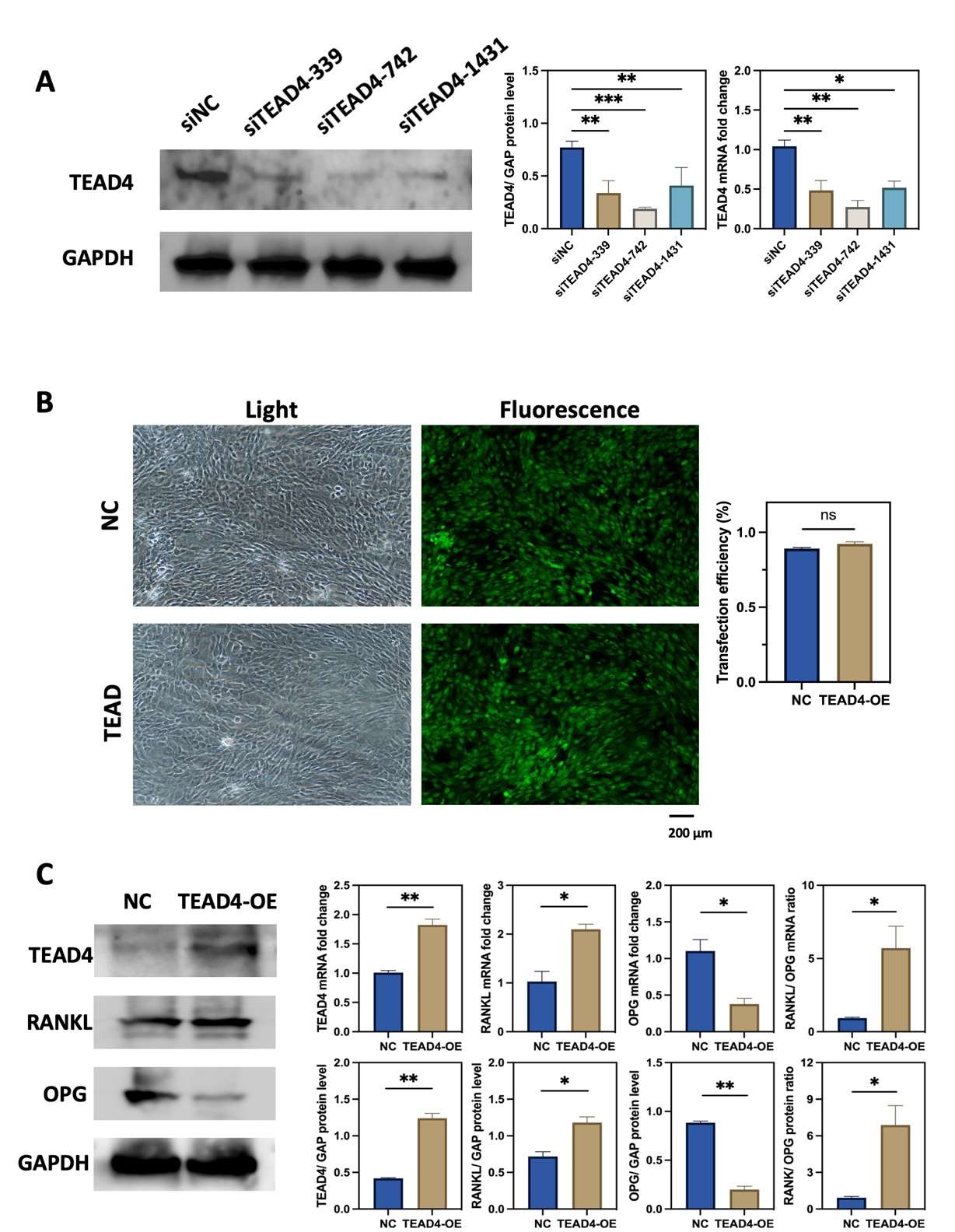


**Figure S4** The knockdown and overexpression of TEAD4 in PDLSCs. A. The knockdown efficiency of siTEAD4. qPCR and western blot showed that siTEAD4-742 possessed the highest knockdown efficiency (n = 3). *P < 0.05, **P < 0.01, ***P < 0.001 vs. siNC. B. Representative fluorescence images of the overexpression of TEAD4 lentivirus. NC was transfected by GFP lentivirus. Quantitative analysis showed that the transfection efficiency of TEAD4 and GFP lentivirus had no significant difference (n = 3). ns: no significant difference vs. NC. Scale bar: 200 μm. C. qPCR and western blot of RANKL and OPG after overexpression of TEAD4 lentivirus in PDLCSs. The protein and mRNA levels of the RANKL/OPG ratio were increased after overexpression of TEAD4 (n = 3). *P < 0.05, **P < 0.01 vs. NC.

| **Table S1.** Top 10 of identified proteins associated with KAT6A | | | | | |
| --- | --- | --- | --- | --- | --- |
| Number | Gene Symbol | Description | Accession | Coverage  (%) | Molecular Function |
| 1 | P4HB | Protein disulfide-isomerase | P07237 | 17 | signal transduction activity or receptor binding; cytoskeletal activity |
| 2 | BASP1 | Brain acid soluble protein 1 | P80723 | 11 | nucleic acid binding activity |
| 3 | **YAP1** | Transcriptional coactivator YAP1 | P46937 | 10 | nucleic acid binding activity |
| 4 | TAGLN | Transgelin | Q01995 | 9 | cytoskeletal activity |
| 5 | S100A6 | Protein S100-A6 | P06703 | 9 | transporter activity; cytoskeletal activity |
| 6 | TXNRD1 | Thioredoxin reductase 1, cytoplasmic | Q16881 | 8 | other molecular function |
| 7 | PGM3 | Phosphoacetylglucosamine mutase | O95394 | 6 | other molecular function |
| 8 | SEPTIN9 | Septin-9 | Q9UHD8 | 6 | other molecular function |
| 9 | NME1 | Nucleoside diphosphate kinase A | P15531 | 6 | translation activity; kinase activity; other molecular function |
| 10 | FARSB | Phenylalanine--tRNA ligase beta subunit | Q9NSD9 | 5 | nucleic acid binding activity; other molecular function |

**T****able S2.** List of primers used in the study.

| Primer | Sense primer (5’-3’) | Antisense primer (5’-3’) |
| --- | --- | --- |
| KAT6A | CC GCCTCAACACTAAGGCAG | GACACGGGAGGTAAACACGAG |
| YAP | TA GCCCTGCGTAGCCAGTTA | TCATGCTTAGTCCACTGTCTGT |
| GCN5 | TA CTAGGGGTCTTCTCGGCTTG | GGATACGTGGTCAGCCAAGG |
| P300 | GG GCAGTGTGCCAAACCAGATG | CATAGCCCATAGGCGGGTTG |
| PCAF | GT CTGCAAGGCCGAGGAGTCT | GAAACATGAGCAGCTAGGGC |
| TEAD4 | GG ACACTACTCTTACCGCATCC | TCAAAGACATAGGCAATGCACA |
| OPG | GT GGCGAATGCAAGGAAGG | CCACTCCAAATCCAGGAGGG |
| RANKL | CA ACATATCGTTGGATCACAGCA | GACAGACTCACTTTATGGGAACC |
| TRAP | GACTGTGCAGATCCTGGGTG | GGTCAGAGAATACGTCCTCAAAG |
| GAPDH | CG ACAGCAGCCGCATCTT | CCAATACGACCAAATCCGTTG |

**Table S3.** List of siRNA sequence.

| siRNA | Sequence |
| --- | --- |
| siKAT6A-1320 | Sense strand GCAUGUGGAUAUGUCAAAUTT |
|  | Antisense strand AUUUGACAUAUCCACAUGCTT |
| siKAT6A-2097 | Sense strand GGUUCCAUCCUCCUGCCAATT |
|  | Antisense strand UUGGCAGGAGGAUGGAACCTT |
| siKAT6A-2388 | Sense strand GCAGGUUUCUCAUCGAUUUTT |
|  | Antisense strand AAAUCGAUGAGAAACCUGCTT |
| siYAP-1536 | Sense strand GACCAAUAGCUCAGAUCCUUU |
|  | Antisense strand AAAGGAUCUGAGCUAUUGGUC |
| siYAP-1676 | Sense strand CAGGUGAUACUAUCAACCAAA |
|  | Antisense strand UUUGGUUGAUAGUAUCACCUG |
| siYAP-1876 | Sense strand CAGGUGAUACUAUCAACCAAA |
|  | Antisense strand UUUGGUUGAUAGUAUCACCUG |
| siTEAD4-339 | Sense strand CCCGGAUAUUGAGCAGAGUUU |
|  | Antisense strand AAACUCUGCUCAAUAUCCGGG |
| siTEAD4-742 | Sense strand CCUUUCUCUCAGCAAACCUAU |
|  | Antisense strand AUAGGUUUGCUGAGAGAAAGG |
| siTEAD4-1431 | Sense strand GCUGUGCAUUGCCUAUGUCUU |
|  | Antisense strand AAGACAUAGGCAAUGCACAGC |
